# Mitochondrial miRNA- miR-181c-5p and mitomiR-106a-5p levels as indicators in cardiovascular disease patients

**DOI:** 10.1007/s11033-026-11617-0

**Published:** 2026-03-25

**Authors:** Ari Nabi, Raya Kh. Yashooa, Tola Abdulsattar Faraj, Karzan Abdulmuhsin Mohammad, Suhad A. Mustafa, Giuseppe Troiano, Mario Dioguari, Abd Al-Bar Al-Farha, Nazzareno Capitanio

**Affiliations:** 1 Department of Clinical and Experimental Medicine, University of Foggia, Foggia, 71122 Italy; 2 Department of Biology, College of Science, Salahaddin University-Erbil, Erbil, 44001 Kurdistan Iraq; 3 Department of Biology, College of Education for Pure Sciences, University of Al- Hamdaniya, Mosul, 41002 Iraq; 4 School of Medicine, British International University, Erbil, Kurdistan Region of Iraq, Erbil, Iraq; 5 Department of Medical Analysis, Faculty of Applied Science, Tishk International University, Erbil, Iraq; 6 Department of Basic Sciences, College of Medicine, Hawler Medical University, Erbil, Iraq; 7 General Directorate of Scientific Research Center, Salahaddin University-Erbil, Kurdistan Region, 44001 Iraq; 8 Department of Biotechnology and Food Sciences, Technical Agricultural College-Mosul, Northern Technical University, Mosul, 41002 Iraq

**Keywords:** mitomiRs, Expression, Cardiovascular, RT- qPCR, miR-181c, miR-106a

## Abstract

**Background:**

Mitochondrial dysfunction is a key contributor to the pathophysiology of cardiovascular disease (CVD), one of the leading causes of morbidity and mortality worldwide. Mitochondria-associated microRNAs (mitomiRs) have emerged as critical regulators of mitochondrial homeostasis and cardiac function; however, their clinical utility as circulating biomarkers remains incompletely defined. This study aimed to evaluate the diagnostic potential of circulating hsa-miR-181c-5p and hsa-miR-106a-5p in patients with CVD and to assess their applicability as early, non-invasive biomarkers in an Iraqi cohort.

**Methods:**

In this case–control study, plasma samples were obtained from 30 patients with clinically diagnosed cardiovascular disease and 30 age-matched healthy controls. Total RNA was extracted, followed by complementary DNA synthesis. Expression levels of hsa-miR-181c-5p and hsa-miR-106a-5p were quantified using reverse transcription quantitative real-time polymerase chain reaction (RT-qPCR). Diagnostic performance was evaluated using receiver operating characteristic (ROC) curve analysis.

**Results:**

Plasma levels of hsa-miR-181c-5p were significantly upregulated in CVD patients, exhibiting a three-fold increase compared with controls (*p* = 0.0001). Likewise, hsa-miR-106a-5p expression was elevated by approximately four-fold in the CVD group (*p* = 0.0008). ROC analysis demonstrated robust discriminatory power for both miRNAs, with an area under the curve (AUC) of 0.796 for hsa-miR-181c-5p (*p* = 0.0002) and 0.749 for hsa-miR-106a-5p (*p* = 0.0010).

**Conclusions:**

Our findings identify circulating hsa-miR-181c-5p and hsa-miR-106a-5p as promising mitomiR-based biomarkers for cardiovascular disease. Their significant upregulation and diagnostic accuracy support their potential role in early, non-invasive detection of CVD and highlight the clinical relevance of mitochondrial miRNA dysregulation in cardiovascular pathology.

**Graphical abstract:**

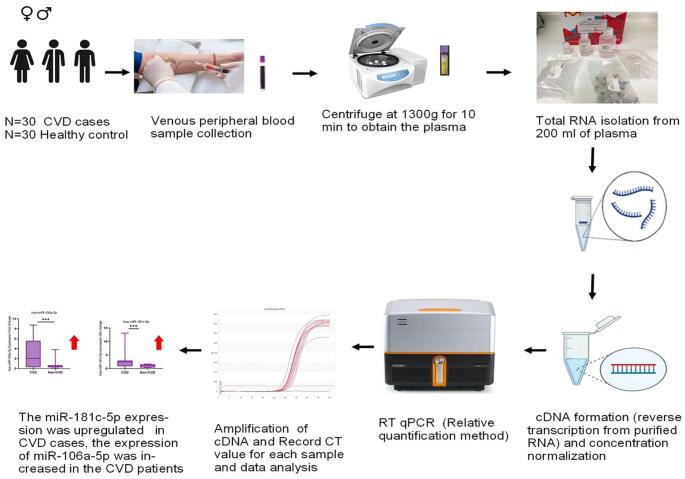

## Introduction

Mitochondria are multifunctional cell organelles that play significant roles in a range of cellular processes, including reactive oxygen species (ROS) generation, energy production, Ca 2 + homeostasis, apoptosis, and cell survival. Mitochondrial activities are also important in cellular homeostasis since they convert energy and produce ATP. These pathways include the electron transport chain (ETC) and the tricarboxylic acid cycle (TAC) [1]. Previous study stresses that mitochondria regulate several signaling pathways that regulate energy balance, metabolism, and activation [2]. Pre-miRNAs and mature miRNAs have a specific position in subcellular structures and organelles such the nucleus, mitochondria, endoplasmic reticulum, and nucleolus, according to research on numerous species of miRNA [2].

MicroRNAs (miRNAs) are small noncoding RNAs (17–23 nucleotides in length). Their function in post-transcriptional regulation of gene expression is crucial by binding to the 3′untranslated region (3′UTR) of the target genes, they mostly impede genetic information translation and/or cause the breakdown of messenger RNA (mRNA), disrupting gene expression [3]. Several different kinds of cells and animals have miRNAs, which were formerly known as “mitomiRs,” and these molecules are found in mitochondria [4]. One way to classify miRNAs occurring in mitochondria is as “mitomiRs.” It is possible that the mitochondrial genome transcribes the miRNAs, or that the nucleus genome encodes them and imports them [3].

CVDs, which affect heart and blood vessel tissues, are the main cause of sickness and mortality worldwide. By 2030, cardiovascular diseases expected to kill around 23.6 million people. The main causes of CVD mortality are heart diseases and stroke [5, 6]. The gene expression was investigated and studied in different diseases. Recent studies indicate that miRNAs along with mitochondria are crucial in the pathogenesis of cardiac diseases. The manipulation of genes by miRNAs is closely associated with mitochondrial function, and miRNAs are pivotal in the initiation and advancement of cardiovascular conditions [7]. Several studies have been shown association between mitomiRs and cardiovascular disease [8–12].

The deregulation of mitomiRs may interfere with and disrupt the homeostasis of mitochondrial and cellular senescence [13]. One example is miRNA-181c, which is produced by nuclear DNA. It has been demonstrated to control the expression and function of mitochondrial genes, thus impacting cellular physiology [14]. Some of the mitomiRs detected to be involved in cardiac diseases include miR-181c-5p and miR-106a-p. miR-181c-5p has received considerable attention due to its unique ability to localize to mitochondria and directly regulate mitochondrial gene expression in cardiomyocytes. Mitochondrial dysfunction is a key hallmark of cardiovascular diseases (CVDs), including heart failure, cardiac hypertrophy, and obesity-associated cardiomyopathy. Previous studies have highlighted that the primary effect of miR-181c upregulation in the cardiovascular system may be mediated through an increase in mitochondrial calcium concentration ([Ca²⁺]m). In cardiomyocytes, Mitochondrial Calcium Uptake 1 (MICU1) and Specificity Protein 1 (Sp1) are important downstream targets of miR-181c [15]. Sp1 is a member of the zinc-finger protein family [22] and plays a crucial role in regulating the expression of numerous genes [23]. Oxidative stress has been shown to modulate Sp1 activity through post-translational modifications, particularly at cysteine residues within its zinc-finger domain [24]. As a result, ROS can regulate Sp1-dependent gene transcription by reducing Sp1 protein levels via post-translational modification. Although overexpression of miR-181c leads to downregulation of both MICU1 and Sp1 proteins, this effect is not due to direct binding of the miRNA seed sequence to the 3′-untranslated regions of MICU1 or Sp1 mRNAs. Instead, miR-181c overexpression alters MICU1 and Sp1 expression indirectly by increasing ROS production through direct interaction with mitochondrially encoded cytochrome c oxidase subunit I (mt-COX1) mRNA [15].

The previous investigation demonstrated that within the same family of microRNAs, only miR-181c is absorbed into the mitochondrial compartment of cardiomyocytes, excluding miR-181a, b, and d [14]. Significantly, miR-181c binds exclusively to the 3’-end of mt-COX1, a transcript of the mitochondrial genome, and modulates mitochondrial function [7]. Additionally, it has been shown that miR-106a has the capacity to target the Mitofusin 2 gene (Mfn2), which plays a crucial role in the regulation of cardiac hypertrophy [15]. Mitofusin 2 is a member of mitofusion protein family, it is positioned in mitochondrial outer membrane and has significant role to maintain mitochondrial structure [16]. Mfn2 is highly expressed in cells and tissues with high metabolic demand, such as the brain, heart, skeletal muscle, liver, and kidney [27]. It facilitates mitochondrial fusion, enabling the exchange of energy and mitochondrial DNA among neighboring mitochondria. In addition, Mfn2 mediates the interaction between the endoplasmic reticulum and mitochondria, thereby supporting mitochondrial respiration and regulating Ca^2+^ transfer [28]. Dysfunction of Mfn2 leads to mitochondrial fragmentation, increased ROS production, altered Ca^2+^-mediated signaling and impaired mitochondrial function, ultimately contributing to pathological cardiac hypertrophy [29].

The recent and previous studies highlighted the protentional role of miR-181c-5p and miR-106a-5p in induce of cardiovascular dysfunction mechanisms [15, 17, 18], while in our region there is no previous studies explores the role of mitomiRs in cardiovascular patients.

This study addresses the diagnostic efficacy of mitomiR-181c-5p and mitomiR-106a-5p, in identifying patients with CVD. It examines the expression of putative free circulating mitomiRNAs in the plasma of patients with cardiovascular diseases and compare it to a group of healthy individuals. This research presents new discoveries on the practical application of mitomiRs as early detection biomarkers for cardiovascular disease in Iraq and the Middle East, addressing the inadequate examination of this topic in the region.

## Materials and methods

### Study participants

The study samples consisted of 60 participants who were selected from private specialized heart clinics in Erbil-city. The samples were divided into two groups: 30 patients diagnosed with cardiovascular disease (CVD) and another 30 persons who were healthy controls (non-CVD). The specimens were gathered from June 2024 to July 2024. Diagnosis was based on the clinical cardiologist and, the criterion of selection of the healthy control group was that the participants did not have any comorbidity; the absence of cardiovascular diseases symptoms in the control group studied was confirmed with the help of ultrasonography.

### Collection of samples and storage

A 3 mL sample of the peripheral blood of each participant in venous blood was kept in its own tubes with anticoagulant EDTA K. The samples were centrifuged to obtain the plasma at 1300 g and 10 min. Afterward the samples subjected to total extraction of RNA and the synthesis of cDNA and then stored at 80^o^ C until they were used for future investigation.

### miRNAs isolation

RNA molecules were isolated from 200 µl plasma using miRNA-specific GenEluteTM Total RNA Kit (Sigma, Aldrich, Germany). The material then was purified, and the DNA was removed using an RNase-Free DNase I kit (Norgen, Biotek Corp, Canada) according to the manufacturer’s instructions. The concentration and purity of the isolated RNAs was measured using a NanoDrop ND-1000 spectrophotometer (Thermo Scientific, Massachusetts, USA).

### Reverse transcription and miRNA polyadenylation

The complementary DNA (cDNAs) were synthesized by reverse transcribing 3µl of the extracted RNA into cDNA. This was performed by using two steps, polyadenylation, where Poly A Polymerase was used to append a poly A tail to the 3’ end of mature miRNA, then reverse-transcribed and annealed with Oligo adaptor primer to mature miRNAs. This has been done through utilizing of the All-in-one miRNA qPCR detection kit (Genecopoeia, USA). The automated thermal cycler PCRmax PCR (TECHNE, UK) was used to carry out the experiments. The samples were incubated in 37 °C over a period of 60 min and the enzymes were deactivated at 85 °C over a period of 5 min. The samples of cDNA were kept at -20 ^o^ C until they were utilized.

### miRNAs selection

**Fig. 1:**
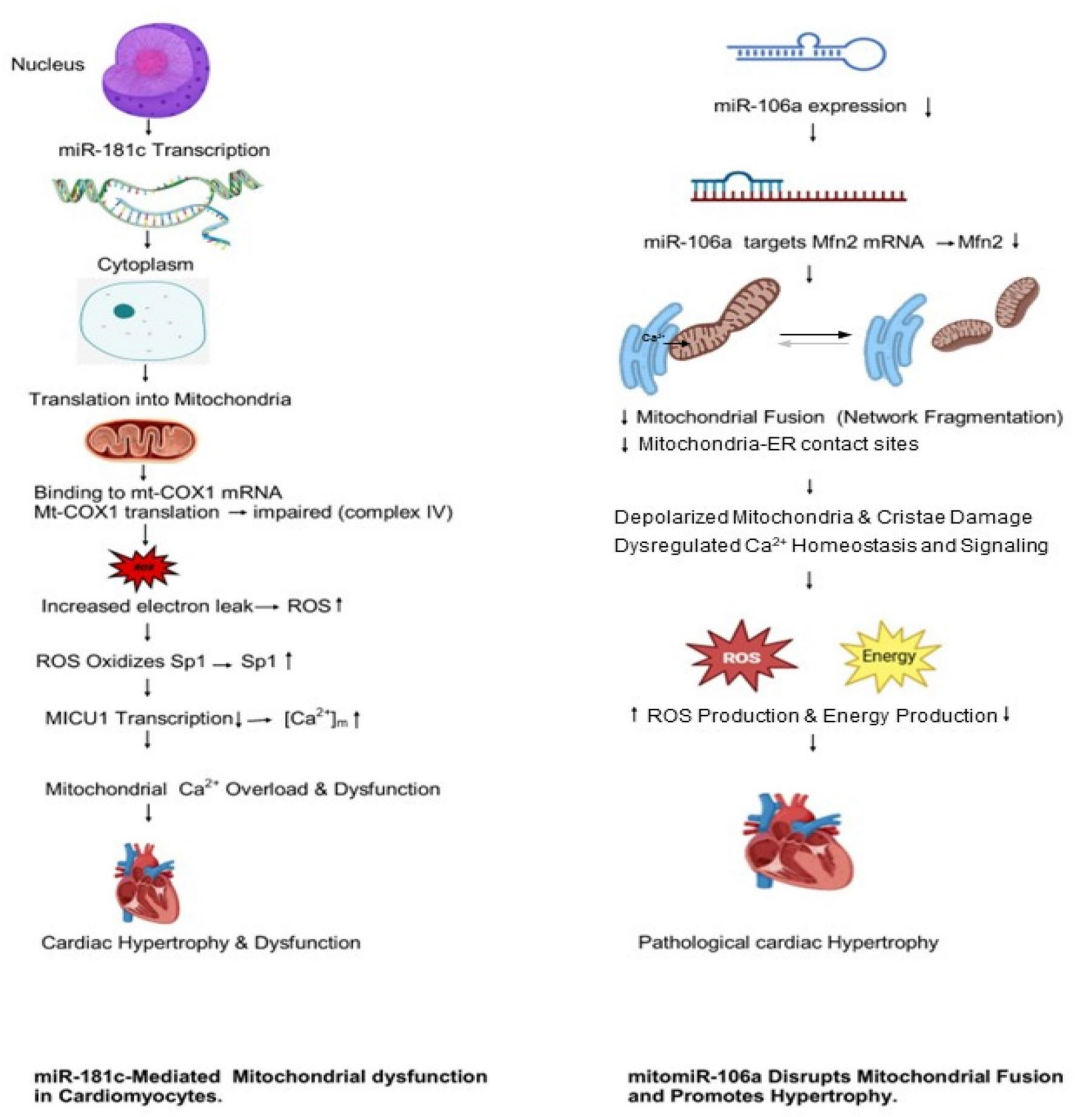
Illustrate the mechanisms of miR-181c and miR-106a in disruption of mitochondrial dysfunction in CVD

The selection of miRNAs was based on an analysis of data from various medical databases, specifically miRBase and TargetScanHuman. The miRNAs hsa-miR-181c-5p and hsa-miR-106a-5p were chosen because of their association with cardiovascular disease (CVD) (Fig. 1).

### Relative quantification of mitomiRs via RT-qPCR

The experiment was conducted by carrying out RT-qPCR in triplicates using the “All-in-one miRNA qPCR detection kit” (Genecopoeia, USA), which contains SYBR^®^ Green. The RT-qPCR was performed by Primer Pro 48 real-time PCR cycler (TECHNE, UK). The RT-PCR primers utilized in Table 1, and a reverse adaptor primer provided by the kit were used to conduct the inquiry. The RNU6-2 gene was used as a control (reference gene) to allow comparison and normalization. Each primer set underwent a two-step quantitative PCR cycling procedure.

**Table 1:** Primers used for the RT-qPCR

Genes	Primer sequences (Forward primers)	Amplicon size (bp)	Catalogue number
Hsa-miR-181c-5p	F:5’- TTCAACCTGTCGGTGAGTAA − 3’	71	HmiRQP0235
Hsa-miR-106a-5p	F:5’-GTGCTTACAGTGCAGGTAGAA − 3’	72	HmiRQP0026
RNU6-2	F:5’- TCGTGAAGCGTTCCATATTTTTAA − 3’	75	HmiRQP9001

The cycling conditions included an initial denaturation stage at 95 °C for 10 min, followed by 50 cycles of denaturation at 95°C for 10 seconds, annealing at 60°C for 20 seconds, and extension at 72°C for 15 seconds. High resolution melting (HRM) curve analysis was conducted by recording the fluorescence intensity at 0.3°C intervals within the temperature range of 65°C to 95°C. The Ct values of each sample were acquired and compared to RNU6-2 using the 2⁻ΔΔCt method. The expression level was assessed adopting the comparative threshold cycle (CT) technique, and the outcomes were evaluated using the 2−∆∆Ct approach. [19, 20].

### Statistical analysis

.Statistical analysis of the data based mainly upon the software packages: SPSS version 25, GraphPad Prism version 9.0.0, and MedCalc software version 18.11.3. The chi-square test of association was applied to compare the proportions of the two groups. The Mann-Whitney test, also referred to as a student's t-test used to compare the data of two groups in cases when the data did not conform to a normal distribution. The receiver operating characteristic (ROC) curve was employed to calculate the area under the curve (AUC) for each miRNA. The purpose was to establish the optimal point at which the screening criteria are most efficient in distinguishing between cardiovascular disease (CVD) and non-CVD and to evaluate the diagnostic significance. A P-value of 0.05 or lower was considered statistically significant. 

## Results

### Characteristics of cohorts and demographic variables

This study examined blood miRNAs in 30 cardiovascular disease patients and 30 healthy controls. The average age of patients in the test group was 73 ± 7.1, whereas in the control group it was 71.9 ± 9.3. No significant age or sex differences existed across groups (Table 2). The statistical analysis (Table 3) found no significant differences in stroke, hypertension, diabetes, smoking, inflammatory disorders, and cancer variables.

**Table 2:** Distinctive characteristics between the CVD and the non-CVD cases

Variables		CVD	Non-CVD	Total	
		No.	No.	No.	*P*- value ^a, b^
		*N* = 30	*N* = 30	*N* = 60	
Age		73 ± 7.1	71.9 ± 9.3		0.5384^b^
Sex	Male	11 37.9%	21 70.0%		0.628^a^
	female	18 62.1%	9 30.0%		
Male/ Female		11/18	21/9		

ͣ Student t-test, ^b^Chi-square, Means ± SD

**Table 3:** Demographic attributes of individuals with CVD and those without CVD, in relation to family history and certain physiological factors

Variables	CVD	Non-CVD	Total	
	No. (%)	No. (%)	No. (%)	*P*-value^a^
**Hypertension**				0.393
Yes	17 (56.6%)	1 (3.3%)	18 (30.0%)	
No	13 (43.3%)	29 (96.3%)	42 (70.0%)	
**History of stroke**				
Yes	6 (20.0%)	0	6 (10.0%)	0.611
No	24 (80.0%)	30 (100%)	54 (90.0%)	
**Diabetes mellitus**				0.947
Yes	8 (26.6%)	7 (11.6%)	15 (25.0%)	
No	22 (73.3%)	23 (76.6%)	45 (75.0%)	
**Smoking history**				0.779
Yes	4 (13.3%)	9 (30.0%)	13 (21.6%)	
No	26 (86.6%)	21 (66.6%)	47 (78.3%)	
**Inflammatory diseases**				0.603
Yes	6 (20.0%)	0	6 (10.0%)	
No	24 (80.0%)	30 (100%)	54 (90.0%)	
**Cancer**				0.782
Yes	2 (6.66%)	0	2 (3.335%)	
No	28 (93.3%)	30 (100%)	58 (96.6%)	
**Total**	30	30	60	

^a^ Chi-square tests

### Quantitation of miRNA expression levels based on qPCR

**Fig. 2:**
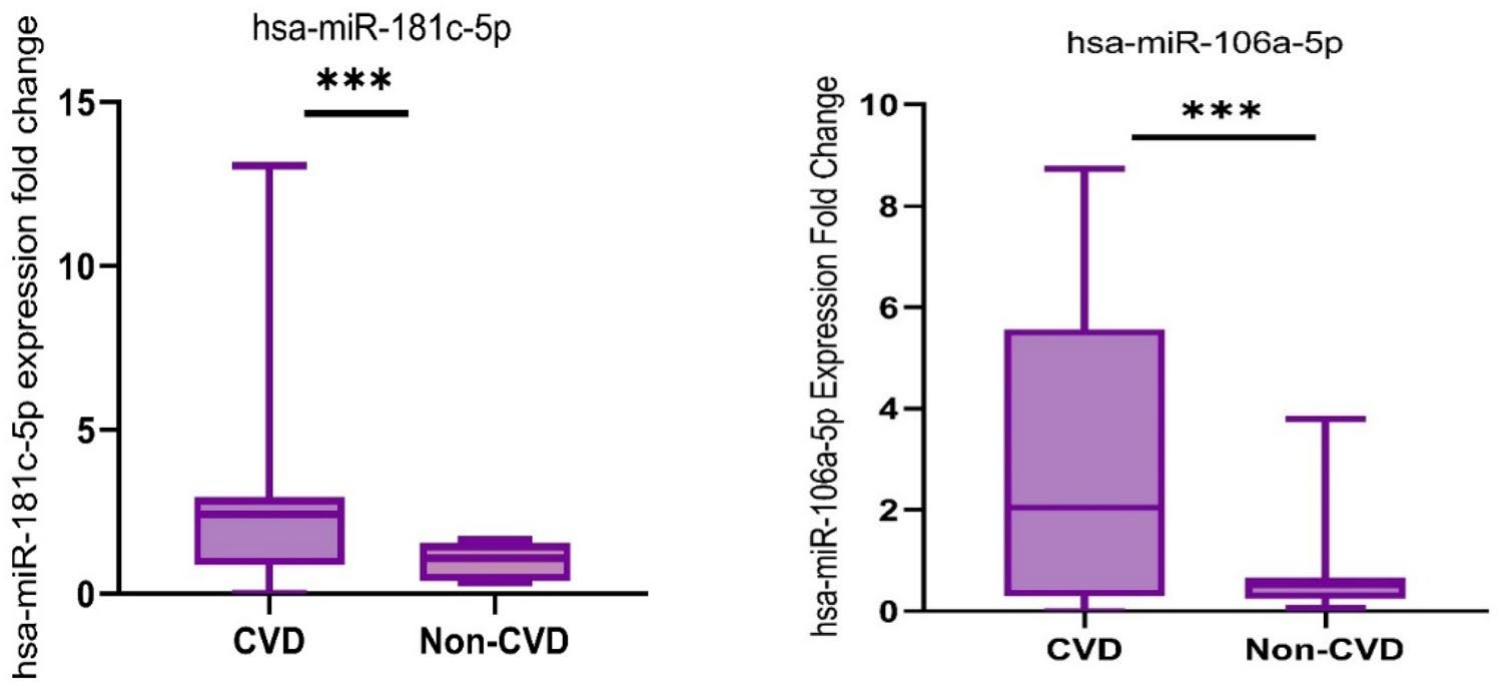
Comparative analysis of the normalized transcript level of has-miR-181c-5p (left panel) and has-miR-106a-5p (right panel) between cardiovascular disease patients (CVD) and healthy subjects (non-CVD). ***, *P* = 0.0001; ***, *P* = 0.0008

A total of sixty samples were obtained from private clinics in Erbil city. These samples were divided into two cohorts: thirty participants diagnosed with cardiovascular disease and thirty healthy individuals serving as the control group. Our analysis showed that the expression level of circulating hsa-miR-181c-5p differed significantly (P-value = 0.0001) between the cardiovascular disease patients and the healthy control group (Table 4; Fig. 2). The mean fold change in gene expression was three times higher in the cardiovascular disease cases compared to the non-cardiovascular disease group.

**Table 4:** The expression level of hsa-miR-181c-5p and hsa-mir-106a-5p in CVD and Non-CVD groups

miRNAs	Number	Diagnosis	medium	Interquartile Range (25%-75%)	*P*-value*
hsa-miR-181c-5p	*n* = 30	CVD	2.423	0.8708–2.942	0.0001
		Non-CVD	1.085	0.3772-1.540	
hsa-miR-106a-5p	*n* = 30	CVD	2.043	0.2975–5.563	0.0008
		Non-CVD	0.5155	0.2542–0.6617	

*Mann-Whitney test

The statistical analysis revealed a substantial difference in the expression level of hsa-miR-106a-5p in plasma between the two groups. The expression of this gene was up-regulated by approximately four times in the mean fold change in the patient with cardiovascular disease compared to the control group (fold change expression). The statistical significance of this difference was determined to be P-value = 0.0008 (see Table 4; Fig. 2).

**Fig. 3:**
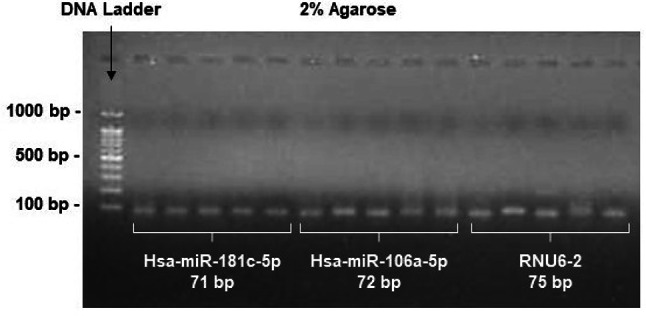
Electrophoretic analysis of the miRNA’s qPCR products following Real-Time PCR

In addition, a positive control provided by Genecopoeia, USA, together with primers, yielded a positive CT value; a non-template control (NTC) was employed as a negative control (NC) to guarantee specificity and validate the results [21]. Further, the NTC was employed to verify the absence of contamination in both the cDNA and qPCR reagent. The gel electrophoresis performed was used to assess the accurate size of the miRNAs qPCR product that we obtained (Fig. 3).

### The diagnostic value of miRNAs

**Fig. 4:**
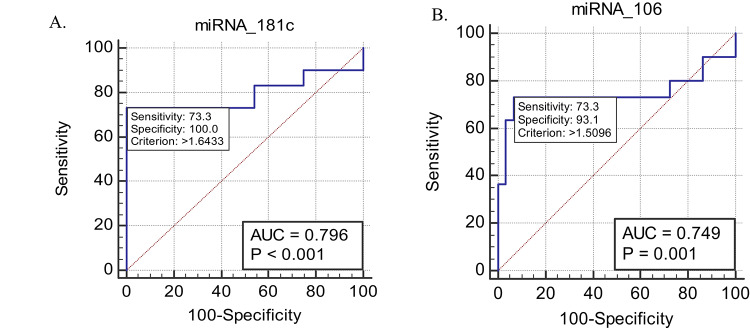
ROC curve study for hsa-miR-181c-5p and 106a-5p to predict and detect cardiovascular disease and distinguish CVD patients from non-CVD persons (**A**) AUC = 0.796. (**B**) Hsa-miR-106a-5p ROC curve, AUC = 0.749

The ROC determined AUC, sensitivity, and specificity. It also examined the diagnostic value of circulating blood hsa-miR-181c-5p and 106a-5p as cardiovascular disease biomarkers. Hsa-miR-181c-5p had an AUC of 0.796 with a 95% CI of 0.6654–0.9263 (Fig. 4A; Table 5). The area under the ROC for hsa-miR-106a-5p was 0.749 (95% CI 0.6073–0.8916) (Fig. 4B; Table 5). We estimated sensitivity and specificity and used the McNamara test to compare cardiovascular patients’ miRNA values to healthy controls (Table 6).

**Table 5:** Area under the curve (AUC) for hsa-miR-181c-p and hsa-miR-106a-5p ROC

“Area Under the Curve (AUC)					
miRNAs	Area	Std. Error	Asymptotic Sig. *P*-value	Asymptotic 95% Confidence Interval	
				Lower Bound	Upper Bound
hsa-miR-181c-5p	0.796	0.066	< 0.001	0.6654	0.9263
hsa-miR-106a-5p	0.749	0.072	0.001	0.6073	0.8916”

**Table 6:** Validity of miR-181c-5p and miR106a5p in predicting cardiovascular disease

“miRNAs	Criterion	Sensitivity	Specificity	PV+	PV-
miR-181c-5p	> 1.643262	73.33	100	100	75
miR-106a-5p	> 1.5096	73.33	93.1	91.7	77.1"

PV- Predicative value negative, PV+ predictive value positive

## Discussion

The miR-181c-5p and miR-106a-5p was selected in this study based on previous work that documented their involvement in regulation of mitochondria and cardiovascular pathophysiology. miR-181c-5p was selected due to it's particular classification as a mitochondria associated microRNA, which the transcription occurs in the nucleus while translocate's into cardiomyocyte mitochondria. Previous investigations have demonstrated that miR-181c-5p directly interacts with the mitochondrial-encoded transcript mt-COX1 (Fig. 1), a crucial element of cytochrome c oxidase (complex IV), leading to altered electron transport chain activity and increased generation of mitochondrial reactive oxygen species (ROS). Mitochondrial oxidative stress and calcium dysregulation are critical contributors to cardiac hypertrophy, heart failure, and obesity-related cardiomyopathy; thus, miR-181c-5p is a biologically significant target for investigating the mitochondrial mechanisms underlying heart disease [17].

Concurrently, mitomiR-106a-5p was selected because of its established role in regulating mitochondrial dynamics, particularly the mitochondrial fusion processes essential for maintaining bioenergetic homeostasis in cardiomyocytes. Scientists have shown that miR-106a-5p selectively aids mitofusin-2 (Mfn2), a vital protein which is found on the outer mitochondrial membrane that is necessary in mitochondrial fusion and structural stability. The up-regulation of miR-106a-5p reduces the level of Mfn2, disrupts the integrity of the mitochondria, completely deforms the mitochondrial functioning process, and raises the generation of ROS, which leads to a considerable condition of ventricular hypertrophy (Fig. 1). The involvement of miR-106a-5p aids cardiovascular disease in analyzing the structural remodelling of mitochondria, which combines with the action of miR-181c-5p. The choice of these two miRNAs helps in the overall assessment of mitochondrial gene regulation, dynamics and stress responses as far as the development of CVD is concerned [15].

Our observations imply that mitomiR-181c-5p and mitomiR-106a-5p can serve as potential biomarkers for detecting CVD. Studies specifically addressing mitochondrial microRNAs are significantly scarce, particularly in Iraq and the Middle Eastern region. CVD has been described as a cause of morbidity and mortality in the world [22], and it is significantly associated with numerous health and economic burdens in recent years. Non-invasive and potential biomarkers for diagnosing and identifying CVD have received potential research attention [23]. Circulating miRNAs serve as a clinical indicator due to the high levels of stability and accessibility. In addition, miRNAs can be readily collected from bodily fluids such as saliva, blood, and urine utilizing non-invasive methods [24]. The miRNAs have been identified as a significant regulators of metabolism [25], and may effect mitochondria by controlling the mitochondrial expression proteins encoded via nuclear genes [26]. Mitochondria have been shown to harbor miRNAs [27], which could potentially impact mitochondrial dysfunction or function [15]. Several previous studies have detected mitomiRs to regulate the function of many cardiac diseases [28, 29]. Abnormal expression of mitomiRs in bodily fluids is correlated with impaired mitochondrial activity. Therefore, the policy of inadequate standardization of the efficient, rapid, dependable, and affordable determination of mitochondrial miRNAs makes the application of circulating mitomiRs as non-invasive markers of clinical utilization merely theoretical. The verification and identification of changes in circulating miRNA expression, specific to certain disease stages, their relationship with aetiology, and the incorporation of multicenter clinical trials may clarify their potential role as biomarkers. The potential combination of circulating mitochondrial miRNAs may serve as a basis for future research [30]. MitomiRs can be transmitted into the bloodstream through various mechanisms. Passive release cellular damage, whether necrotic or inflammatory, can promote the passive release of mitomiRs into the extracellular environment and subsequently into the bloodstream. Active secretion via extracellular vesicles (EVs): Cells are known to actively release mitomiRs through exosomes and microvesicles. This procedure serves as a regulated mechanism for the release of mitomiR. MitomiRs can be released into circulation through blood vessels, accompanied by protein complexes such as Argonaute (AGO) proteins, which safeguard them from degradation [31].

In the cohort of CVD patients, included in the present study, we did not find significant differences in variables regarding age, gender, hypertension, history of stroke, diabetes mellitus, smoking history, inflammatory diseases, and cancer, which may be due to the low number of participants that used in this study as in a previous meta-analysis conducted by Leng et al. [32] who found no significant difference in CVD in different genders [32]. However, a study performed by the American Heart Association emphasizes that blood pressure, stroke, diabetes, smoking, physical activity, weight, and diet contribute to CVD health [33].

Our statistical analysis detected the expression of hsa-miR-181c-5p was up-regulated in the CVD group compared to healthy individuals. In addition, the ROC curve analysis emphasizes that hsa-miR-181c-5p is a potential diagnostic marker in identifying cardiovascular disease. Studies emphasize that elevating the has-miR-181c-5p level in CVD patients contributes to the effect on the function and structure of the heart consisting of dysfunction of mitochondria, apoptosis, inflammation, endothelial dysfunction, and ROS production [17, 24, 34, 35]. In a study conducted by Das [36] showed that miR-181c, originating from the nuclear genome, transfers to the mitochondria and performs a significant role in modifying mitochondrial function and controlling gene expression. They have shown that miR-181c controls the expression of the mt-COX1, which is a catalytic subunit of (complex IV) of the respiratory chain, by binding to its 3’-end. Furthermore, they revealed that the overexpression of miR-181c leads to a reduction in mt-COX1 protein and function, alteration of (complex IV) structure, and indirect elevation of ROS generation [36]. Moreover, it was previously identified that miR-181c is expressed significantly in obesity and indirectly increases the activity of the calcium transporter Micu1 by promoting the production of ROS through its interaction with mt-Cox1 [17]. Elevated levels of ROS modify the function of the transcription factor Sp1, which works as a positive regulator of Micu1. These alterations ultimately result in an elevation of mitochondrial calcium levels, leading to the development of heart hypertrophy [17]. In addition, miR-181c-5p triggers programmed cell death of heart muscle cells by hypoxia/reoxygenation influence [35]. Several studies have demonstrated the clinical significance of miR-181c-5p: the study conducted by [37] found overexpression in patients that have heart disease that matched our finding. Another work performed via [24] utilized a mouse model that detected the up-expression of miR-181c-5p and down-expression in Sirtuin 1 (SIRT1), resulting in myocardial pathology. In addition, they revealed down-regulation of miR-181c-5p and up-regulation of SIRT1 leading to decreased acetylated p65 protein levels and a change in cardiac apoptosis [24]. Similar results by Gevaert et al. [38] showed increasing circulating miR-181c-5p level in heart disease cases consistent with our finding [38].

Compared to the non-cardiovascular group, the cardiovascular patients had an enhanced level of has-miR-106a-5p, as shown in the present research. In addition, the results of the ROC curve study demonstrated that hsa-miR-106a-5p has the ability to detect CVD. One of the key regulators of angiogenesis is miR-106a, which is a member of the miR-17 family [39]. In addition, research has shown that miR-106a inhibits atherosclerosis-related VEGFA production, which in turn inhibits the formation of new blood vessels [40]. Reduced oxidative stress-induced endothelial cell damage is a major result of targeting STAT3 by downregulating miR-106a-5p [41]. The hypertrophic features produced by AngII were shown to be fully reversible upon miR-106a reduction. One of the proteins that miR-106a directly targets is Mfn2, which is known to have a role in mitochondrial fusion and is therefore important for mitochondrial functioning. Overexpression of Mfn2 rendered miR-106a’s hypertrophic characteristics ineffective, while the (reduction) lack of Mfn2 rendered miR-106a inhibitors hypertrophic [15]. In addition, the defects in mitochondrial cristae shape, mitochondrial membrane depolarization, and an increase in ROS generation caused by sustained cardiac stress were successfully repaired by either miR-106a suppression or Mfn2 overexpression [15]. Damage to cells, mitochondrial malfunction, and cell death are all consequences of oxidative stress, which is an important contributor to atherosclerosis progression [42]. Damaged angiogenesis was associated with atherosclerosis-related miR-106a-5p overexpression in conditions where the LDL receptor was absent in both chromosomes [40]. Our finding agrees with other results reported by Zhang and his colleagues [30] who detected that the overexpression of miR-106a-5p in atherosclerosis cases correlated with declining the target Mfn2 expression [30]. The same result by Hao [43] emphasize our finding, which detected elevated the expression level of miR-106a-5p in heart disease cases. In previous study it was detected that the overexpression of FGD5-AS1could bind miR-106a-5p suggesting it as a therapeutic target for myocardial H/R injury reducing inflammation and cell apoptosis [43].

The inapplicability of any similar research has necessitated the fact that more in-depth research is done to establish the validity of these findings using larger cohorts of demographically different people. Investigations of mitomiRs in the pathophysiology underlying CVD provide a clue of avenues of early diagnosis and application of specific therapies, particularly in settings where the occurrence of CVD is on the increase.

## Conclusion

The study conducted to reveals novel results on the functions and role of the mitochondrial microRNA miR-181c-5p and mitomiR-106a-5p as a diagnostic marker of the CVD. The current research seeks to fill the knowledge gap on the topic of mitochondrial microRNAs practiced in Iraq. The mitomiRs are differentially expressed in cardiovascular disease patients, which detected that the mitomiRs may serve as biomarkers to provide the early diagnosis and assessment of the diseases. Further extensive clinical investigations are required to establish their relevance in CVD, as well as to be able to underline their molecular requirements in CVD development. The investigation of mitochondrial microRNAs may lead to the development a novel, non-invasive therapeutic and diagnostic strategies in the field of cardiovascular medicine.

## Data Availability

No datasets were generated or analysed during the current study.
